# Medial knee joint contact force in the intact limb during walking in recently ambulatory service members with unilateral limb loss: a cross-sectional study

**DOI:** 10.7717/peerj.2960

**Published:** 2017-02-02

**Authors:** Ross H. Miller, Rebecca L. Krupenevich, Alison L. Pruziner, Erik J. Wolf, Barri L. Schnall

**Affiliations:** 1 Department of Kinesiology, University of Maryland, College Park, MD, United States; 2 Neuroscience & Cognitive Science Program, University of Maryland, College Park, MD, United States; 3 Walter Reed National Military Medical Center, Bethesda, MD, United States; 4 DoD-VA Extremity Trauma and Amputation Center of Excellence, Bethesda, MD, United States; 5 Department of Rehabilitation, Uniformed Services University of the Health Sciences, Bethesda, MD, United States

**Keywords:** Load, Gait, Prosthesis, Transtibial, Transfemoral, Osteoarthritis, Military

## Abstract

**Background:**

Individuals with unilateral lower limb amputation have a high risk of developing knee osteoarthritis (OA) in their intact limb as they age. This risk may be related to joint loading experienced earlier in life. We hypothesized that loading during walking would be greater in the intact limb of young US military service members with limb loss than in controls with no limb loss.

**Methods:**

Cross-sectional instrumented gait analysis at self-selected walking speeds with a limb loss group (*N* = 10, age 27 ± 5 years, 170 ± 36 days since last surgery) including five service members with transtibial limb loss and five with transfemoral limb loss, all walking independently with their first prosthesis for approximately two months. Controls (*N* = 10, age 30 ± 4 years) were service members with no overt demographical risk factors for knee OA. 3D inverse dynamics modeling was performed to calculate joint moments and medial knee joint contact forces (JCF) were calculated using a reduction-based musculoskeletal modeling method and expressed relative to body weight (BW).

**Results:**

Peak JCF and maximum JCF loading rate were significantly greater in limb loss (184% BW, 2,469% BW/s) vs. controls (157% BW, 1,985% BW/s), with large effect sizes. Results were robust to probabilistic perturbations to the knee model parameters.

**Discussion:**

Assuming these data are reflective of joint loading experienced in daily life, they support a “mechanical overloading” hypothesis for the risk of developing knee OA in the intact limb of limb loss subjects. Examination of the evolution of gait mechanics, joint loading, and joint health over time, as well as interventions to reduce load or strengthen the ability of the joint to withstand loads, is warranted.

## Introduction

Since 2001, over 1,600 United States military service members have sustained traumatic injuries involving major limb loss (Fischer, 2015). Individuals with unilateral lower limb loss have a high risk of developing secondary physical conditions later in life, including osteoarthritis (OA) in their intact limb (Gailey et al., 2008; Morgenroth, Gellhorn & Suri, 2012). In veterans with unilateral limb loss, the prevalence of knee OA is 30%–90% greater in the intact limb compared to veterans without limb loss (Hungerford & Cockin, 1975; Lemaire & Fisher, 1994; Norvell et al., 2005). Most of the subjects in these previous studies were older adults who had been living with their amputations for several decades. Recent reviews have indicated a rising incidence of idiopathic knee OA in the young military population (Showery et al., 2016) and in service members with limb loss specifically (Farrokhi et al., 2016). The younger service members with limb loss from recent conflicts may therefore live with a relatively high risk of developing knee OA for many years.

Our long-term goal is to develop interventions that can be implemented early after limb loss to minimize the risk of developing knee OA later in life. Achieving this goal is challenging because the causal mechanisms of OA are unknown. However, mechanical loading is suspected to play a major role in the disease’s etiology (Andriacchi & Mündermann, 2006; Maly, 2008; Felson, 2013), and overloading the intact limb by deliberately or subconsciously favoring it during activities of daily living is a long-standing hypothesis for explaining the prevalence of knee OA in the limb loss population (Borgmann, 1960). The fatigue life of human articular cartilage *in vitro* suggests that the stresses from repetitive loading in walking could produce mechanical failure of the superficial collagen fibers well within the human lifespan (Weightman, Chappell & Jenkins, 1978; Bellucci & Seedhom, 2001). Relatedly, the “cartilage conditioning” hypothesis argues that cartilage *in vivo* adapts to withstand frequently encountered stress levels (Seedhom, 2006). If abrupt changes in gait mechanics due to amputation and prosthesis use result in sudden increases in loading of the intact limb, these loads could overwhelm the adaptive response of cartilage, particularly if it has recently been weakened due to a long period of unloading from injury, surgery, and recovery. For example, knee cartilage glycosaminoglycan content, which affects the compressive stiffness of cartilage, remains below baseline for at least a year following six weeks of immobilization in humans (Owman et al., 2014). Similar results are seen in animal models (Jurvelin et al., 1986). The time when unilateral limb loss patients first begin walking again could therefore be a particularly important time to assess their joint loading.

In this study, we therefore examined knee joint loading in the intact limb of relatively young service members with unilateral limb loss who had recently begun walking independently with their prostheses for the first time. We tested the hypothesis that loading of the medial knee joint, as indicated by the peak, loading rate, and impulse of the medial joint contact force, is greater during self-paced walking in the intact limb of young service members with limb loss than in a control group of similar age and background (young service members) without limb loss. These three outcome variables were chosen because they have all previously been associated with knee OA risk, and it is unknown which is most important. The medial knee was chosen because the knee is the most common site of OA (Centers for Disease Control and Prevention, 2015), and because medial knee OA is more common than lateral knee OA (Wise et al., 2012). An elevated risk of general knee OA has been reported in young military service members with limb loss (Farrokhi et al., 2016). The risk of medial knee OA specifically in this population is unknown, but bone mineral density and joint structure suggest a high risk for medial knee OA in this population (Royer & Koenig, 2005; Morgenroth et al., 2014).

Recent studies suggest that the peak external knee adduction moment (KAM), the most widely-used metric for quantifying medial knee joint loading in gait (Foroughi, Smith & Vanwanseele, 2009; Simic et al., 2011), is similar in the intact limb of young service members with limb loss vs. controls (Pruziner et al., 2014; Esposito & Wilken, 2014), and that including the external knee flexion moment (KFM) more accurately estimates medial joint loading than using the KAM alone (Manal et al., 2015). We therefore elected to quantify medial knee joint loading using a model that considers the KAM and the KFM as well as the timing of muscle activity within the gait cycle that contributes to these moments (Schipplein & Andriacchi, 1991).

## Materials & Methods

### Subjects

The study design was cross-sectional, with a “limb loss” group and a “control” group. The limb loss group consisted of 10 service members with unilateral limb loss. Five subjects had transtibial amputations and five had transfemoral amputations. The descriptive statistics of the limb loss group (mean ± SD) were: age 27 ± 5 years, height 1.77 ± 0.05 m, mass 81.1 ± 18.4 kg, and 170(36) days from their most recent amputation-related surgery. All subjects were male and had been walking independently without assistive devices other than their prosthesis for an average of two months at the time of data collection. Additional inclusion criteria were no previous diagnosis of OA, no pain during activities of daily living greater than 4 on a 10-point scale, no limb loss elsewhere on the body, no history of traumatic injury to the intact limb, and no history of traumatic brain injury or other medical issues known to affect gait.

The control group consisted of 10 male service members with no limb loss and similar descriptive statistics to the limb loss group (age 30 ± 4 years, height 1.79 ± 0.07 m, mass 83.8 ± 14.3 kg), who also met all the inclusion criteria. Walter Reed National Military Medical Center granted ethical approval to carry out the study within its facilities (IRB reference number 350985). All protocols were approved by the ethics committee. All subjects were briefed on the study protocols and gave informed written consent prior to participating.

### Experimental setup

An instrumented gait analysis was performed while subjects walked across a level 15-m walkway. Subjects wore shorts and their own athletic footwear. The limb loss subjects used their own clinically prescribed passive prosthesis. Positions of 23 retroreflective markers on the pelvis and lower limbs were sampled at 120 Hz using 23 optical motion capture cameras (Vicon, Oxford, UK). Ground reaction forces (GRF) were sampled synchronously at 1,200 Hz using six force platforms (AMTI, Watertown, MA, USA) embedded in the walkway. Individual markers were attached by double-sided tape on the anterior- and posterior-superior iliac spines, iliac crests, greater trochanters, medial and lateral femoral epicondyles, medial and lateral malleoli, heads of the 2nd and 5th metatarsals, and heel of the shoe. Lightweight shells with clusters of four markers were attached to the thigh and shank using elastic wraps. The medial markers were removed after a standing calibration trial and were reconstructed as virtual markers during the walking trials.

### Protocol

Subjects walked along the walkway at self-selected speed and cadence. Instructions were to walk in a “normal and comfortable” fashion. Each subject walked back and forth along the walkway until five acceptable trials were collected, with “acceptable” defined as each foot contained entirely within the bounds of a single force platform and both feet never simultaneously contacting the same platform.

### Data processing

Marker positions and GRF from each trial were exported to Visual3D (C-Motion, Germantown, MD, USA) for further analysis. Marker positions and GRF were smoothed using a 4th-order dual-pass Butterworth filter with cutoff frequencies of 6 Hz and 50 Hz, respectively. A linked-segment model of each subject’s pelvis and intact limb was defined using marker positions from a standing calibration trial. The hip joint center was estimated from the positions of the pelvis markers (Bell, Brand & Peterson, 1989). The knee center was estimated as the midpoint of the femoral condyle markers, and the ankle joint center was estimated as the midpoint of the malleoli markers. The long axes of the thigh and shank were defined between the proximal and distal joint centers. The frontal plane axis for both segments was defined from the cross-product of the long axis and the vector between the femoral condyles. The sagittal plane axis was the cross-product of the frontal plane and long axes. Segment tracking during gait trials was calculated from the positions of marker clusters on rigid shells.

Joint angles during gait were calculated using 6DOF pose estimation, with a Cardan *Xyz* rotation sequence (Wu & Cavanagh, 1995). Resultant joint forces and moments were calculated by iterative Newton–Euler inverse dynamics beginning at the foot (Selbie, Hamill & Kepple, 2014). The resultant knee forces and moments were expressed in the shank reference frame.

**Figure 1:**
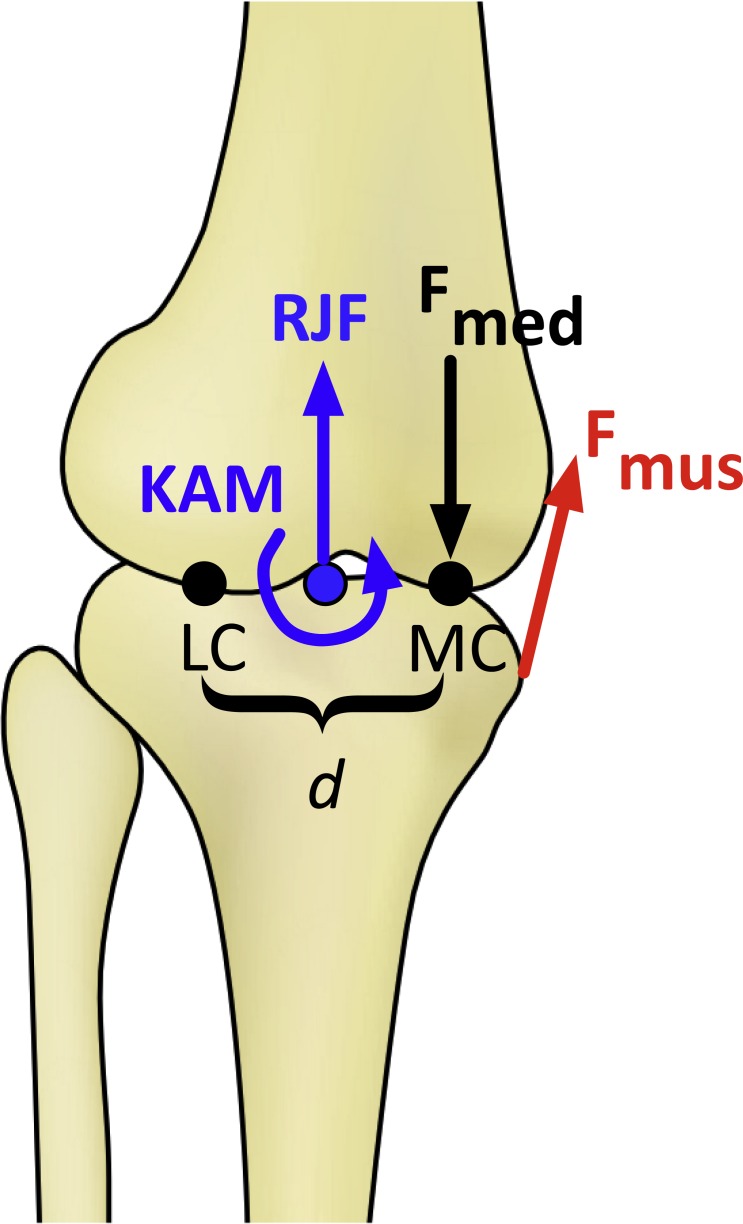
Schematic of the knee model in the frontal plane for calculating the medial knee joint contact force (F_med_). KAM, knee adduction moment; RJF, resultant axial joint force; F_mus_, muscle force, determined by the knee flexion moment; LC and MC, medial and lateral contact points, separated by distance *d*. F_med_ is calculated by balancing the moments produced about the point LC (Schipplein & Andriacchi, 1991).

### Joint contact force modeling

Medial knee joint contact forces were calculated using the model of Schipplein & Andriacchi (1991). Muscle moment arms and orientations were defined as quadratic functions of the knee flexion angle using average values for men from Wretenberg et al. (1996). The KFM was assumed to be produced by the quadriceps if the moment was extensor, by the hamstrings if the moment was flexor in swing or during early stance, and by the gastrocnemius if the moment was flexor in late stance. Forces in the individual hamstrings muscles (biceps femoris, semimembranosus, semitendinosus) and the two gastrocnemius heads were distributed by the ratios of their physiological cross-sectional areas from Arnold et al. (2010). The medial contact force was then calculated by balancing the frontal plane moments about the lateral contact point (Fig. 1). Cruciate and collateral ligament forces were included in the contact force calculation using the method described by Morrison (1968). The distance between the medial and lateral tibiofemoral contact points in the frontal plane was assumed to be 5.0 cm on average and was scaled linearly for each subject by the distance between the medial and lateral femoral condyle markers during the standing calibration trial. Baseline model parameters are summarized in Table 1.

Note that this model assumes zero antagonistic co-contraction. This assumption could potentially underestimate contact forces around heel-strike, when the quadriceps and hamstrings are both active (Sutherland, 2001). However, since knee muscle co-contraction in early stance is similar between limb loss subjects and controls (Seyedali et al., 2012), this assumption does not bias the results in favor of the hypothesis.

### Statistical analysis

The planned comparisons were the peak, loading rate, and impulse of the contact force between groups. These outcome variables were scaled by bodyweight (BW), with the mass of the prosthesis included in this calculation for the limb loss subjects. Loading rate was defined as the maximum loading rate during 10%–90% of the time from heel-strike until the first peak.

Results will be presented for the transtibial and transfemoral subjects separately. However, due to the small sample sizes, these subjects were combined into a single limb loss group for statistical comparison with the control group. It will be seen that the differences in contact forces between the limb loss and control groups were not driven by the transtibial or transfemoral subjects specifically (i.e., contact forces were similar on average for transtibial and transfemoral subjects).

Normality of the outcome variables was assessed using Kolmogorov–Smirnov tests. All tests passed at the *α* = 0.05 level. Subsequently, comparisons between groups were made using independent Student’s *t*-tests (*α* = 0.05, *β* = 0.20) with a False Discover Rate adjustment for the multiple outcome variables. The tests were one-tailed due to the directional nature of the hypothesis. 95% confidence intervals (CI) were also calculated. As an additional conservative check due to the small sample sizes, differences were reported only if the effect size was large (Cohen’s *d* > 0.80). Effect sizes for between-subjects differences in external knee adduction moment (a common surrogate for medial joint loading) and knee OA initiation and progression are typically much smaller than 0.8 (e.g., Amin et al., 2004; Miyazaki et al., 2002), so the requirement of a large effect size is likely a fairly conservative check.

**Table 1:** Medial joint contact force model parameters. PCSA is physiological cross-sectional areas. The three values shown for each moment arm and each muscle angle are values at (0, −30, −60) degrees of knee flexion, respectively, with 0 degrees defined as full extension. Muscle angles are clockwise from the tibial plateau (anterior-positive and lateral-positive). Moment arms and muscle angles were defined as second-order polynomials fit to these data.

	Value	
**PCSA (cm^**2**^)**		Arnold et al. (2010)
Biceps femoris	16.8	
Semimembranosus	19.1	
Semitendinosus	4.9	
Lateral gastrocnemius	9.9	
Medial gastrocnemius	21.4	
**Sagittal moment arms (mm)**		Wretenberg et al. (1996)
Biceps femoris	(−21.5, −22.9, −24.4)	
Semimembranosus	(−35.6, −37.6, −41.3)	
Semitendinosus	(−25.6, −26.4, −31.2)	
Lateral gastrocnemius	(−38.7, −41.0, −47.0)	
Medial gastrocnemius	(−37.9, −40.4, −47.6)	
Patellar tendon	(50.8, 50.6, 44.1)	
**Frontal moment arms (mm)**		Wretenberg et al. (1996)
Biceps femoris	(48.8, 48.4, 48.6)	
Semimembranosus	(−33.5, −33.7, −30.1)	
Semitendinosus	(−29.7, −30.5, −26.3)	
Lateral gastrocnemius	(19.4, 18.4, 19.2)	
Medial gastrocnemius	(−8.6, −15.1, −17.1)	
Patellar tendon	(4.7, 6.7, 9.1)	
**Sagittal muscle angles (deg)**		Wretenberg et al. (1996)
Biceps femoris	(89.6, 88.9, 89.3)	
Semimembranosus	(107.5, 100.3, 98.7)	
Semitendinosus	(105.6, 96.9, 96.6)	
Lateral gastrocnemius	(73.7, 65.6, 61.3)	
Medial gastrocnemius	(74.0, 67.9, 64.3)	
Patellar tendon	(63.8, 58.5, 56.2)	
**Frontal muscle angles (deg)**		Wretenberg et al. (1996)
Biceps femoris	(102.0, 100.6, 100.9)	
Semimembranosus	(84.2, 83.8, 85.3)	
Semitendinosus	(84.7, 89.1, 89.4)	
Lateral gastrocnemius	(85.7, 84.7, 86.1)	
Medial gastrocnemius	(83.9, 82.0, 78.8)	
Patellar tendon	(101.3, 98.0, 95.7)	
**Distance b/w femoral condyles (cm)**	5.0	Terzidis et al. (2012)

### Sensitivity analysis

The knee model (Fig. 1) required input parameters for muscle moment arms, orientations, and physiological cross-sectional areas, and the distance between the tibiofemoral contact points. The necessary imaging data to define these parameters on a subject-specific basis were not available, and the same generic parameter values were used for all subjects except for the contact point distance. In such situations, probabilistic approaches are useful for assessing the sensitivity of model output to parameter value uncertainty (Valero-Cuevas et al., 2009). To assess the sensitivity of the contact force results (and the conclusions drawn from them) to these parameter values, standard normal distributions were formed for each parameter with the nominal value as the mean and a coefficient of variation of 10%, which is a reasonable estimate of the typical variation in these parameters in a homogenous adult male population (Hasson & Caldwell, 2012). The contact force variables were then re-calculated for each subject using parameters randomly drawn from these distributions, and the statistical analysis was performed again. This process was repeated iteratively until the fraction of iterations with significantly greater outcome variables in the limb loss group changed by under 1% over 100 further iterations. The output of this analysis was the fraction of perturbed parameter sets for which the outcome variable in question (peak, loading rate, or impulse) was greater in the limb loss group, from which the sensitivity of the outcome variables to the assumed model parameters could be judged.

## Results

**Figure 2:**
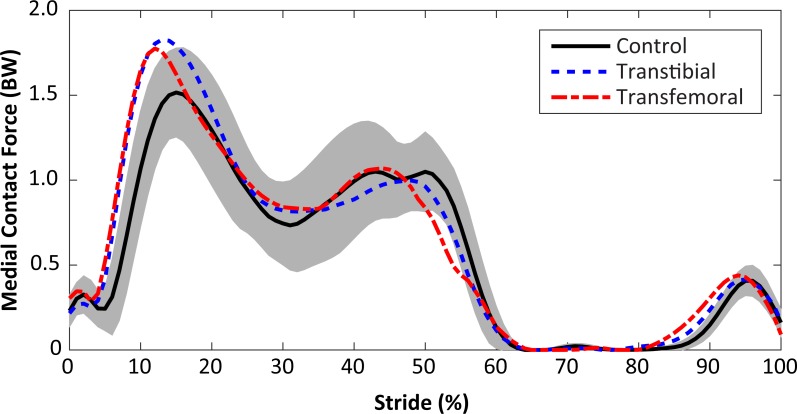
Medial knee joint contact forces in percent bodyweight (BW) during the stride, beginning at heel-strike. Solid, dashed, and dash-dotted lines are means for control, transtibial, and transfemoral subjects. The shaded areas are ±one between-subjects standard deviation for the control subjects.

**Figure 3:**
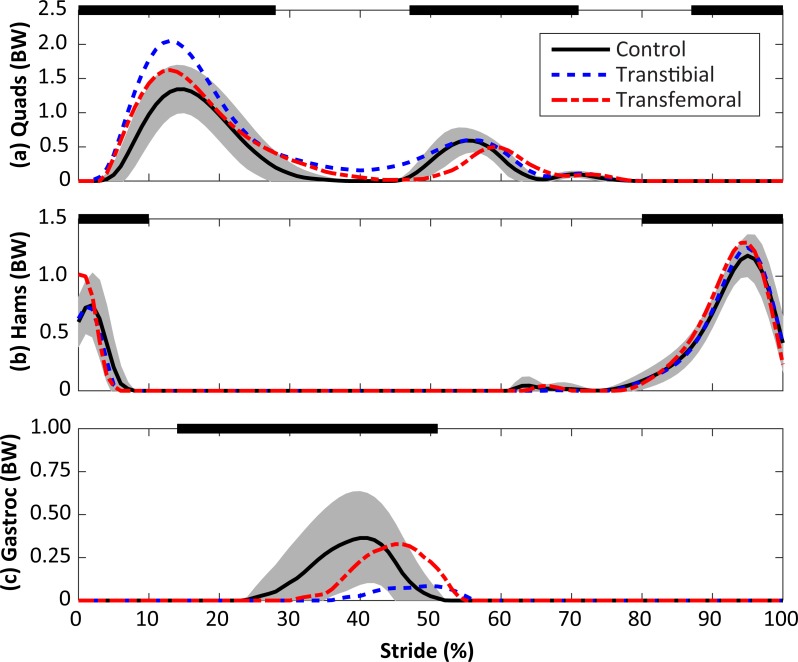
Calculated muscle forces for the quadriceps (Quads, A), hamstrings (Hams, B), and gastrocnemius (Gastroc, C) muscles during the stride, beginning at heel-strike. Solid, dashed, and dash-dotted lines are means for control, transtibial, and transfemoral subjects. The shaded areas are ±one between-subjects standard deviation for the control subjects. Scaling factors were bodyweight (BW). The black bars along the top of each panel denote the fraction(s) of the gait cycle when this muscle group is “on” according to normative electromyograms (Sutherland, 2001), which are similar for the intact limb in limb loss subjects (Seyedali et al., 2012).

Subject-specific data including descriptors, outcome variables, and waveforms of knee joint kinetics, kinematics, and medial contact forces, are included in Data S1. The self-selected walking speeds were similar between groups (1.25 ± 0.19 m/s for limb loss, 1.31 ± 0.10 m/s for controls, *p* = 0.40, *d* = 0.39, 95% CI [−0.19–0.07] m/s). Stride durations were also similar between groups (1.16 ± 0.07 s for limb loss, 1.12 ± 0.07 s for controls, *p* = 0.24, *d* = 0.55, 95% CI [−0.02–0.10] s). The average medial knee joint contact force waveforms are shown for the transtibial, transfemoral, and control subjects in Fig. 2. The contact forces showed the typical two-peaked pattern seen in instrumented knee replacement studies of older adults without limb loss (Walter et al., 2010; Kutzner et al., 2013; Meyer et al., 2013). For the control subjects, the peak force occurred in early stance and averaged 1.57 ± 0.26 BW, which is within the range of values reported in these studies (1.25–2.20 BW). With the exception of a lack of quadriceps activity in late swing, which did not affect the contact force outcome variables, the muscle forces predicted by the model for the quadriceps, hamstrings, and gastrocnemius (Fig. 3) were consistent with normative electromyogram timing for these muscles (Sutherland, 2001; Seyedali et al., 2012).

**Figure 4:**
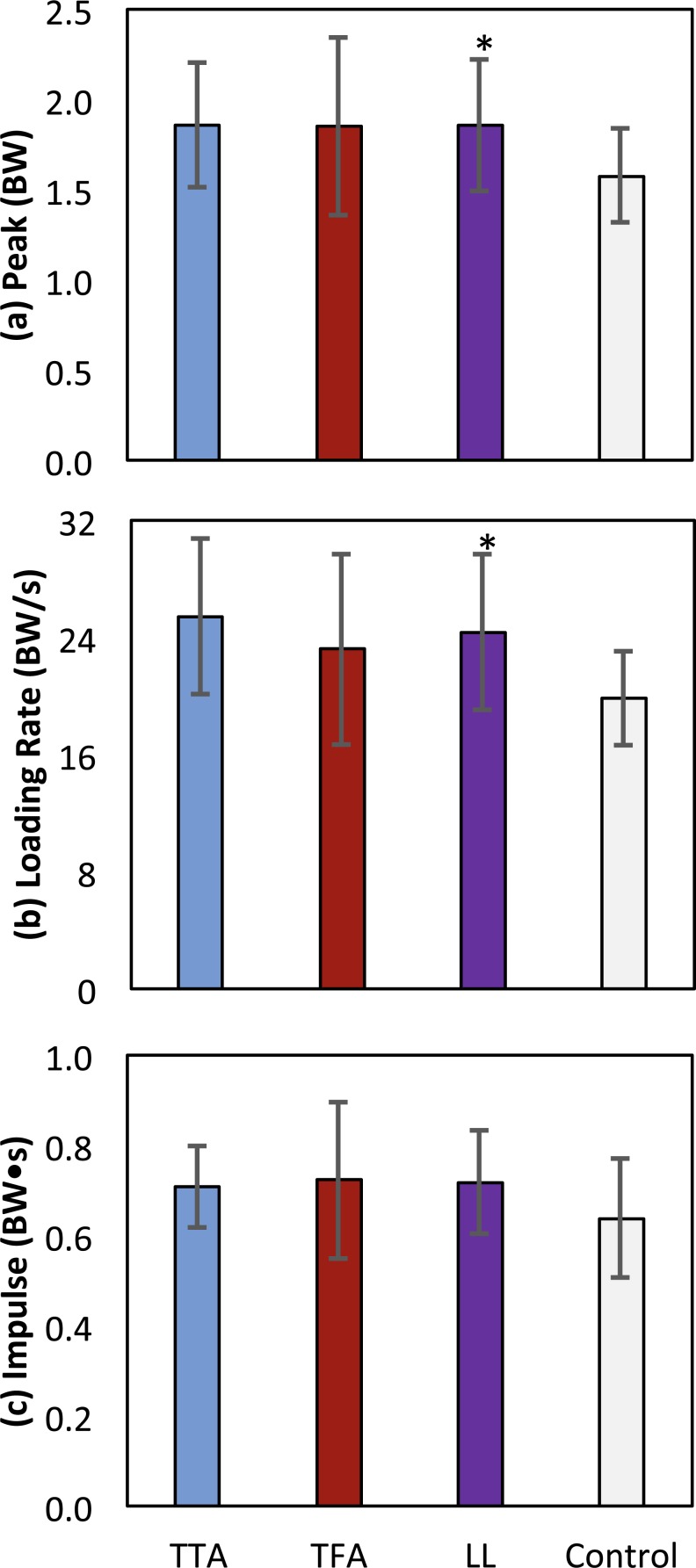
Means ± one between-subjects standard deviation for peak (A), loading rate (B), and impulse (C) of the medial knee joint contact force for the transtibial subjects (TTA), the transfemoral subjects (TFA), and the control subjects. The limb loss bars (LL) are data for the TTA and TFA subjects combined. *, greater than control, with a large effect size (*d* > 0.80).

**Figure 5:**
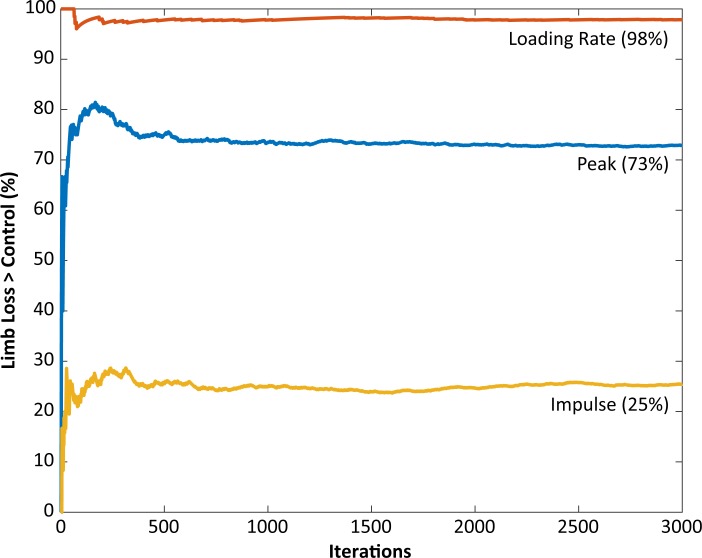
Monte Carlo simulation results for knee model parameter perturbations. The vertical axis shows the fraction of iterations for which the medial joint contact force outcome variable was significantly greater in the limb loss group vs. the control group. The results using the original (unperturbed) parameters are not included here.

The peak contact force was greater in the limb loss group than in the control group (1.84 ± 0.37 vs. 1.57 ± 0.26 BW, *p* = 0.037, *d* = 0.85, 95% CI [−0.01–0.55] BW). Maximum loading rate was also greater in the limb loss group (24.7 ± 5.4 vs. 19.9 ± 3.2 BW/s, *p* = 0.012, *d* = 1.10, 95% CI [1.0–8.7] BW/s). Impulse had a moderate effect size between groups, but were not significantly greater in the limb loss group (0.72 ± 0.12 BW s for limb loss, 0.64 ± 0.13 BW s for controls, *p* = 0.084, *d* = 0.64, 95% CI [−0.03–0.19] BW s). Outcome variables are summarized in Fig. 4. The sensitivity analysis converged after about 3,000 iterations (Fig. 5). The loading rate, peak, and impulse were greater in the limb loss group than in the control group (*p* < 0.05, *d* > 0.80) for 98%, 73%, and 25% of these iterations, respectively.

**Figure 6:**
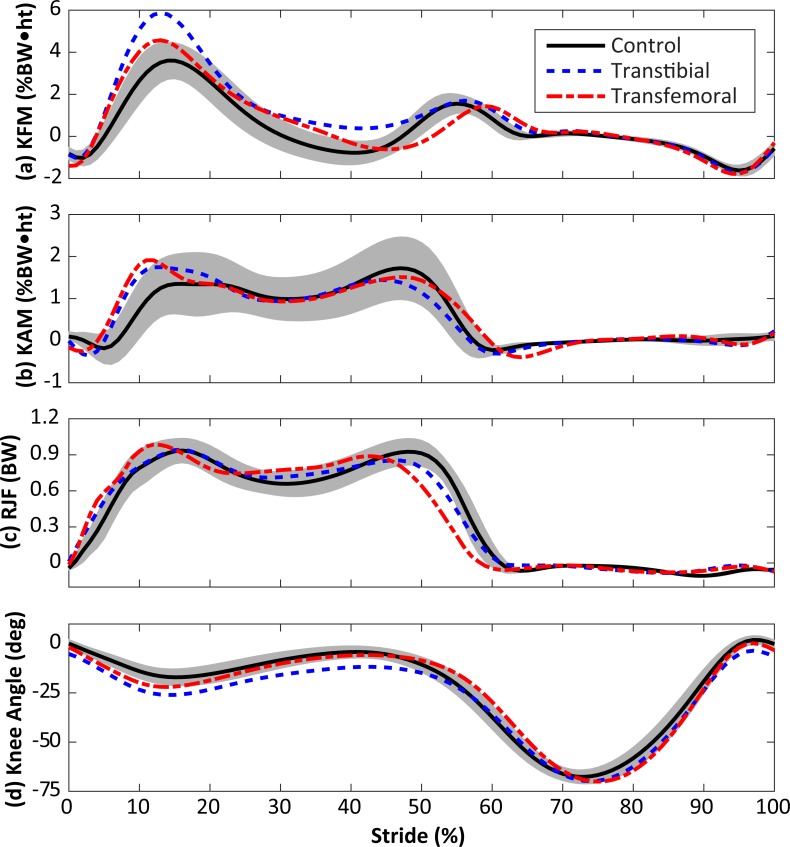
Knee flexion moment (KFM, A), knee adduction moment (KAM, B), resultant joint force along the long axis of the shank (RJF, C), and knee flexion angle (D) during the stride, beginning at heel-strike. Solid, dashed, and dash-dotted lines are means for control, transtibial, and transfemoral subjects. The shaded areas are ±one between-subjects standard deviation for the control subjects. Scaling factors were bodyweight (BW) and height (ht).

The KAM and KFM were not analyzed statistically due to concerns over multiple comparisons with small sample sizes, and the fact that both variables were considered in the calculation of contact forces, but their mean profiles are presented for completeness in Fig. 6. Limb loss subjects tended to have greater peak KFM and KAM than the control subjects, and the transtibial subjects tended to have greater peak KFM than the transfemoral subjects. The primary mechanism by which the transtibial and transfemoral subjects had similar peak contact forces (Fig. 2) despite greater KFM in the transtibial subjects was greater axial resultant joint force in the transfemoral subjects during early stance (Fig. 6).

## Discussion

In this study we tested the hypothesis that knee joint loading during walking is greater in the intact limb of US military service members with unilateral limb loss who are relatively young, recently ambulatory, and otherwise healthy, compared to the limbs of service members with similar demographics and no limb loss. Based on the nominal contact force results (Fig. 4) and the probabilistic analysis of model parameters (Fig. 5), we accept this hypothesis with a high degree of confidence based on the loading rate of the medial joint contact force, and with a moderate degree of confidence based on the peak of the medial joint contact force. Impulse of the contact force did not appear to be greater in the limb loss group.

Before discussing the implications of these results, we first comment on some limitations. The study included small sample sizes, with a mix of transtibial and transfemoral subjects (five each) in the limb loss group. However, the transtibial and transfemoral subjects had similar average height, mass, and self-selected walking speeds (differences of 0.4 cm, 2.8 kg, and 0.02 m/s, respectively), and it can be seen from Fig. 4 that they also had similar contact force results, suggesting that combining these sub-groups into one group was reasonable for the purposes of this study. The limb loss population is difficult to study in large numbers, and we were working with a particular subset of this population (young service members and some fairly restrictive additional inclusion criteria). Due to the small sample size, we took a conservative approach to reporting differences between groups even after adjusting for multiple comparisons, with the requirement of a large effect size. The issues of the knee model parameters and antagonistic co-contraction have already been addressed: these modeling issues may affect the numerical values of the results, but would be unlikely to change the conclusions (Fig. 5). The knee contact model itself (Fig. 1) is a Morrison (1968)-type reduction approach. These models are on the lower end of complexity among the range of musculoskeletal models used for this purpose, but have a long history in biomechanics (Morrison, 1968; Schipplein & Andriacchi, 1991; DeVita & Hortobágyi, 2001; Messier et al., 2011; Willy et al., 2016). History/popularity alone do not validate the approach, but this approach produces similar muscle forces to more mathematically intensive static optimization methods (Kernozek, Gheidi & Ragan, 2016) and knee contact forces in good agreement with instrumented knee replacement measurements (Willy et al., 2016).

The time point at which the gait data were obtained from the limb loss group (shortly after they became independently mobile) could be viewed as a limitation since the gait mechanics of these individuals may change in the future. Although the knee kinetics in the present limb loss subjects (Fig. 6) are similar to those in studies on more experienced prosthesis users (Royer & Koenig, 2005; Esposito & Wilken, 2014), the data here may not represent the “typical” or “average” loads these subjects will experience later in life, due for example to motor learning, experience, changes in fitness, or use of different prostheses. However, the focus on joint loading early on in the rehabilitation process can also be viewed as a strength of the present study. Human articular cartilage appears to undergo at least some degree of structural and functional atrophy in the absence of mechanical loading, and it is unclear if these changes are fully reversible (Vanwanseele et al., 2002; Hudelmaier et al., 2006; Souza et al., 2012; Owman et al., 2014). When new prosthesis users first begin walking independently, their joints have likely undergone a period of at least several weeks with no or minimal mechanical loading following injury, surgery, and recovery. At this early time, we speculate that placing abnormal loads on the intact limb may be particularly dangerous for the future health of the knee. To minimize this risk, we suggest that long periods of unloading should be avoided to the extent that doing so is safe and feasible for the patient.

To the knowledge of the authors, the present study is the first to show that medial knee joint contact forces were greater in the intact limb than in controls. A recent forward dynamics simulation study showed a similar result for the total joint contact force during walking in individuals with unilateral transtibial limb loss (Silverman & Neptune, 2014). Knee OA has a rising incidence among young United States military service members over the past 10 years, and there is a need to develop more effective preventive strategies in at-risk sub-groups of this population (Showery et al., 2016). There are presently no longitudinal studies on baseline joint loading and the initiation of knee OA in the limb loss population. However, Morgenroth et al. (2014) found that the KAM peak, impulse, and loading rate were all significantly correlated with the degree of knee structural abnormality present in the intact limb of middle-aged adults (mean age 56 years) with unilateral transfemoral amputations. The present results suggest that relatively high loads are present on the medial knee of the intact limb when young service members with limb loss begin to walk independently, and when interpreted in light of Morgenroth et al. (2014), that the long-term consequence of these loads may be structural degeneration of the knee. These suggestions are in need of verification in longitudinal studies.

Relatedly, while the present results suggest medial knee joint loading was greater in the limb loss group, the size of the “minimum meaningful difference” that actually affects the risk for knee OA is unknown. Studies using the external knee adduction moment suggest that effect sizes for differences in medial joint loading during walking and the initiation and progression knee OA in older adults may be small (Amin et al., 2004; Miyazaki et al., 2002), but it is unknown if this suggestion generalized to actual medial joint contact forces or to a younger military limb loss population. Two recent studies suggest that the “minimum detectable change” in medial knee joint loading from gait modification is about 0.25–0.30 BW for peak and about 0.04 BW s for impulse (Gardinier et al., 2013; Barrios & Willson, 2016). Those data were from within-subject designs, where the present data are between-subjects, but they suggest that differences smaller than these values may be difficult to reliably detect in gait analysis, even if they are biologically meaningful. For reference, the average differences between the limb loss and control results in the present study were 0.27 BW for peak and 0.08 BW s for impulse. Additional knowledge from longitudinal studies is needed to understand which features of joint loading and cartilage mechanics are most important for predicting future structural degeneration, and if critical thresholds for those variables exist.

As noted earlier, the KAM is presently the most popular variable for assessing medial knee joint loading in human gait. While we did not analyze the KAM statistically due to concerns over the small sample sizes and multiple comparisons, visual inspection of the KAM (Fig. 6) suggests that similar conclusions would have been reached had we used the KAM rather than the medial joint contact force as the primary outcome variable: greater peak and greater loading rate in the limb loss group. However, we caution that this result was likely coincidental and is not a mechanical requirement. The KAM alone does not dictate the loading of the medial knee, as recent instrumented knee implant studies have shown (Walter et al., 2010; Kutzner et al., 2013; Meyer et al., 2013). Relatedly, the KFM has a major influence on the shape, magnitude, and medial/lateral ratio of joint contact forces, and should be considered when assessing joint loading in gait (Manal et al., 2015).

## Conclusions

In summary, the present results suggest that young, recently ambulatory service members with unilateral limb loss place relatively high loads on their medial knee when walking compared to controls without limb loss. We suggest these loads may be a risk factor for future development of knee OA, a common secondary condition in this population. Further longitudinal study and development of preventive interventions (e.g., physical activity guidelines, prosthesis designs) is warranted. The results also indicate that knee contact model parameter values can be an important consideration in cross-sectional studies. Here we investigated the overall sensitivity (perturbing all contact model parameters simultaneously), but sensitivity to particular parameters of interest may be a relevant topic for future work or specific applications.

##  Supplemental Information

10.7717/peerj.2960/supp-1 Data S1 Subject descriptive characteristics, contact forces, and outcome variables

## Additional Information and Declarations

### Data Availability

The following information was supplied regarding data availability: The raw data has been supplied as a Supplementary File.
